# Inhibition of the Wnt Signalling Pathway: An Avenue to Control Breast Cancer Aggressiveness

**DOI:** 10.3390/ijms21239069

**Published:** 2020-11-28

**Authors:** Lorenzo Castagnoli, Elda Tagliabue, Serenella M. Pupa

**Affiliations:** Molecular Targeting Unit, Department of Research, Fondazione IRCCS Istituto Nazionale dei Tumori di Milano, Via Amadeo 42, 20133 Milan, Italy; lorenzo.castagnoli@istitutotumori.mi.it (L.C.); elda.tagliabue@istitutotumori.mi.it (E.T.)

**Keywords:** Wnt pathway, breast cancer, cancer stem cells, epithelial-mesenchymaltransition, resistance to therapy

## Abstract

Breast cancer (BC) is the most common tumour in women. Although the introduction of novel therapeutic approaches in clinical practice has dramatically improved the clinical outcome of BC patients, this malignant disease remains the second leading cause of cancer-related death worldwide. The wingless/integrated (Wnt) signalling pathway represents a crucial molecular node relevantly implicated in the regulation of normal somatic stem cells as well as cancer stem cell (CSC) traits and the epithelial–mesenchymal transition cell program. Accordingly, Wnt signalling is heavily dysregulated in BC, and the altered expression of different Wnt genes is significantly associated with cancer-related aggressive behaviours. For all these reasons, Wnt signalling represents a promising therapeutic target currently under clinical investigation to achieve cancer eradication by eliminating CSCs, considered by most to be responsible for tumour initiation, relapse, and drug resistance. In this review, we summarized the current knowledge on the Wnt signalling pathway in BC and have presented evidence implicating the suitability of Wnt targeting in an attempt to improve the outcome of patients without affecting the normal somatic stem cell population.

## 1. Introduction

Breast cancer (BC) is the most frequent female cancer diagnosed in Western countries. In 2018, 404,920 cases of BC were diagnosed in Europe, and approximately 98,750 patients are estimated to have died [1]. Worldwide, approximately 2.1 million cases were diagnosed in 2018, and even if the BC death rate continues to decline, BC is responsible for the greatest number of cancer-related deaths among women [2].

Over the past two decades, several studies have shown that BC is a morphologically, genetically, and clinically heterogeneous group of diseases classified into different molecular subtypes with peculiar biological features associated with variations in treatment response and disease-specific outcomes [3,4]. Although different molecular BC subtypes are characterized by distinctive gene expression profiles and pathobiological features, it was demonstrated that several cancer-related gene pathways are involved in the regulation of BC cell activities among the different subgroups [5]. In particular, the Wnt pathway is involved in many physiological processes and pathological events, and alterations in this signalling have been critically implicated in the regulation of some key biological features of both normal mammary stem cells (NMSCs) and cancer stem cells (CSCs) [6,7]. Indeed, aberrant Wnt signalling governing CSC-related unlimited self-renewal and pluripotency properties is a crucial player in the initiation/maintenance of many cancers by affecting tumour development, cell differentiation, progression, disease relapse, metastasis and drug-resistance processes [8,9,10]. Furthermore, much evidence has underlined a relevant association of dysfunctional Wnt gene pathway activation with other different BC-associated aggressive hallmarks, i.e., evasion of antitumour immune attack [10,11,12], survival [10], cancer metabolism [13,14], and cell motility [15], through the transcriptional control of the target genes involved in such processes [16]. Taking into consideration the major role of Wnt signalling in BC, a relevant challenge consists of designing therapeutic approaches to target its dysregulated activity in breast CSCs (BCSCs) to achieve cancer eradication while being safe enough to not damage the NMSCs necessary for tissue homeostasis and repair [16]. This review focuses on aspects of altered Wnt signalling mechanisms observed in BC, reporting their implications for BC biology and prognosis, and summarizes current progress on targeting the Wnt pathway to optimize the clinical management of BC patients.

## 2. Wnt Signalling Components and Pathways

**Figure 1:**
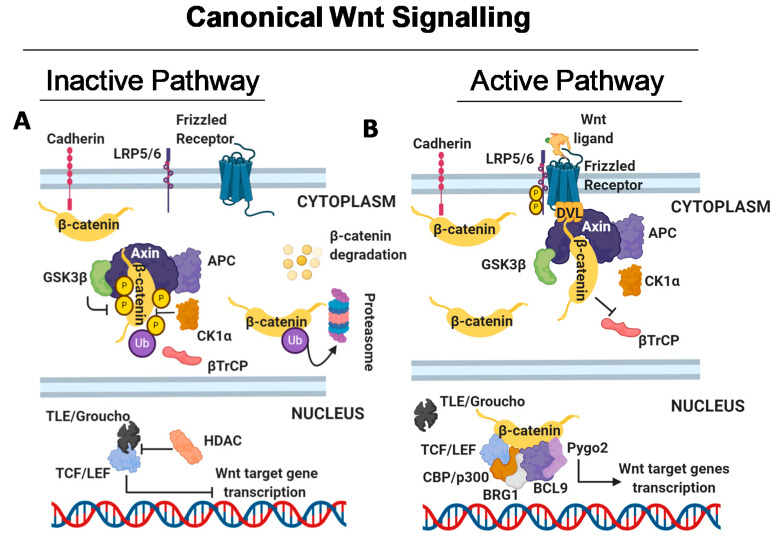

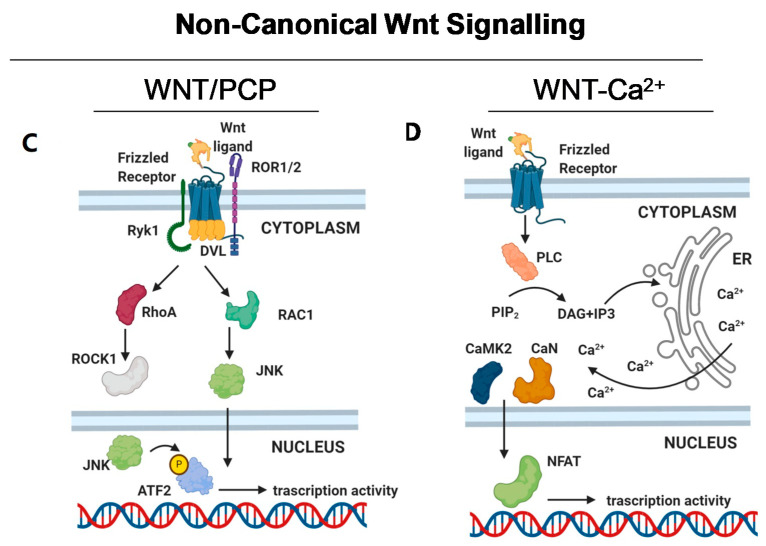
Overview of canonical and non-canonical Wnt signalling. (**A**) In the canonical inactive wingless/integrated (Wnt) pathway, the absence of WNT ligands triggers the phosphorylation of β-catenin via the destruction complex consisting of Axin, adenomatous polyposis coli (APC), and the two kinases glycogen synthase kinase 3β (GSK3β) and casein kinase 1α (CK1α). In this biological scenario, β-catenin is phosphorylated by GSK3β and CK1α, ubiquitinated by β-transducin (β-TrCP), and targeted for proteasomal degradation, which impairs its nuclear translocation. In the absence of nuclear β-catenin, a repressive complex, consisting of T-cell factor/lymphoid enhancer factor (TCF/LEF) and transducin-like enhancer of split (TLE)/Groucho, recruits histone deacetylase (HDAC) to halt Wnt target gene transcription. (**B**) The canonical active Wnt pathway is activated upon binding of WNT ligands to 15 Frizzled (FZD) receptors and LRP5/6 co-receptors. In this state, the destruction complex is recruited to the WNT/receptor complex and inactivated via the dissociation of all its components. This allows the stabilization and accumulation of cytoplasmic β-catenin, which then translocates into the nucleus where it forms an active complex with T-cell factor/lymphoid enhancer factor (TCF/LEF) proteins by displacing TLE/Groucho complexes. Histone modifying co-activators, such as CREB-binding protein (CBP)/p300, Brahma Related Gene 1 (BRG1), B-cell CLL/lymphoma 9 (BCL9) and Pypgpus family PHD finger2 (Pygo2), are recruited, thus allowing the transcription of the Wnt target genes. (**C**) In non-canonical Wnt/planar cell polarity (PCP) signalling, WNT ligands bind to the Receptor Tyrosine Kinase Like Orphan Receptor (ROR)/FZD complex, thus recruiting and activating Dishevelled (DVL). This triggers activation of the small GTPases Ras homolog family member A (RhoA) and Ras-related C3 botulinum toxin substrate 1 (RAC1), which in turn activate Rho Associated Coiled-Coil Containing Protein Kinase (ROCK) and c-Jun N-terminal Kinase (JNK), leading to rearrangements of the cytoskeleton and transcriptional program via activating transcription factor 2 (ATF2). (**D**) Wnt/Ca^2+^ signalling is initiated by G-protein triggered phospholipase C (PLC), which converts phosphatidylinositol 4,5-bisphosphate (PIP2) into diacylglycerol (DAG) and inositol trisphosphate (IP3). This leads to intracellular calcium fluxes and, in turn, activation of calmodulin kinase 2 (CaMK2) and calcineurin (CaN) proteins to induce downstream calcium-dependent cytoskeletal modifications and transcriptional activity via the activating factor nuclear factor of activated T-cell (NFAT).

The human Wnt family is composed of nineteen different secreted cysteine-rich glycoproteins acting as ligands that can participate in one or more Wnt signalling cascades in an autocrine or paracrine manner and more than 15 Frizzled (FZD) family receptors or low-density lipoprotein receptor-related proteins 5/6 (LRP5/6) coreceptors [17]. The pathway is generally divided into three subpathways: canonical (β-catenin-dependent), noncanonical planar cell polarity (PCP) and noncanonical Wnt/calcium (Ca^2+^) cascades [7] (Figure 1A–D).

While the complex consisting of Wnt, FZD, and LRP proteins activates the canonical Wnt/β-catenin-dependent signalling cascade (Figure 1A,B), the complex formed by FZD and/or Receptor Tyrosine Kinase Like Orphan Receptor 1/ Receptor Tyrosine Kinase Like Orphan Receptor 2/Receptor Like Tyrosine Kinase/ (ROR1/ROR2/RYK) activates noncanonical Wnt signalling cascades (the Wnt/PCP and Wnt/Ca^2+^ pathways) (Figure 1C,D).

### 2.1. Canonical Wnt Signalling

Canonical Wnt signalling is activated by the binding of Wnt protein ligands to FZD receptor family proteins, defined by conserved structural features including seven hydrophobic domains and a cysteine-rich ligand-binding domain [18], and to the coreceptors LRP5/6 to initiate a downstream signalling cascade via β-catenin nuclear translocation [7].

#### 2.1.1. Wnt Signalling Off

In the absence of ligands, the Wnt pathway is inactive, and the effector molecule β-catenin is degraded and rendered inactive in the cytoplasm. Specifically, β-catenin is phosphorylated by the multiprotein destruction complex consisting of the scaffolding protein Axin, the tumour suppressor adenomatous polyposis coli (APC), the glycogen synthase kinase 3β (GSK3β), and the casein kinase 1α (CK1α). These proteins have different roles in the regulation of β-catenin phosphorylation specifically at Ser 33 and 37 by the activity of GSK3β protein [19]. Following its phosphorylation, β-catenin is recognized by β-transducin (β-TrCP), a protein that is a part of a ubiquitin ligase complex, ubiquitinated and degraded by proteasome action [20]. Thus, β-catenin degradation prevents its translocation into the nucleus, where a complex formed by T-cell factor/lymphoid enhancer factor (TCF/LEF) and transducin-like enhancer of split (TLE)/Groucho binds histone deacetylases to halt transcription of target genes [20] (Figure 1A).

#### 2.1.2. Wnt Signalling On

In the active Wnt pathway, binding of Wnt ligands to the FZD receptors and LRP5/6 coreceptors triggers the subsequent recruitment of downstream signal mediators. Specifically, LRP receptors are then phosphorylated by CK1α and GSK-3β, which recruit Dishevelled (DVL) proteins to the plasma membrane where they polymerize and are activated [21]. The DVL polymers inactivate the destruction complex, causing its dissociation and leading to the accumulation in the cytoplasm of free β-catenin, which then translocates into the cell nucleus. Nuclear β-catenin displaces TLE/Groucho repressors, forming an active molecular complex with TCF/LEF proteins that bind coactivators such as CREB-binding protein (CBP)/p300, Brahma Related Gene 1 (BRG1), B-cell CLL/lymphoma 9 (BCL9) an Pygopus family PHD finger2 (Pygo2) [22] to transcriptionally activate downstream Wnt target genes, thus promoting uncontrolled cell proliferation [7,10] (Figure 1B).

#### 2.1.3. Canonical Wnt Signaling in CSCs, Therapy Resistance and Immunoevasion

As previously stated, growing evidence has demonstrated that a dysfunctional Wnt signalling cascade is associated with tumour development and progression [23,24] as well as with a variety of human cancers via overexpression or amplification of the growth-promoting activity driven by c-Myc [25,26]. In addition, the Wnt signalling cascade induces the expression of different genes implicated in the regulation of CSC features, such as self-renewal, pluripotency and migration. In particular, in the context of BC, the activation of Wnt/β-catenin signalling is positively correlated with the expression of CSC biomarkers such as cluster of differentiation 44 (CD44) and aldehyde dehydrogenase (ALDH), thereby promoting tumour growth and systemic dissemination [27]. Furthermore, the β-catenin/TCF/LEF transcriptional complex is also involved in the development of chemoresistance, another crucial feature associated with CSC activity. In particular, the expression of ATP-binding cassette subfamily B member 1 (ABCB1), an ATP-dependent drug efflux pump mediating the extrusion of chemotherapy drugs outside the cytoplasm of cancer cells in the extracellular microenvironment, is regulated by β-catenin/TCF/LEF-binding sites in BC [28]. Wnt-target genes also include molecules involved in the dormancy process, a mechanism strictly related to clinical relapse after an initial therapeutic response. In particular, Wnt signalling activation determines the upregulation of CXCR4 and CXCL12, two key molecules that contribute to survival and dormancy in BC cells [29]. Moreover, recent evidence underlined the possibility that Wnt signalling is associated with the insurgence of the immunosuppressive environment in triple-negative breast cancer (TNBC), a cluster of heterogeneous diseases that do not express oestrogen receptor (ER), progesterone receptor (PgR) and epidermal growth factor receptor-2 (HER2) and are characterized by a poor prognosis and lack of targeted treatments. Specifically, our research group reported that TNBC stem cells are characterized by higher expression of programmed death-ligand 1 (PD-L1), the primary ligand of programmed cell death protein expressed on the cell membrane of T-cells, and are able to mediate negative regulation of their anticancer effects in comparison with bulk cells [12]. In this context, the Wnt/β-catenin axis is crucial in the regulation of the dolichyl-diphosphooligosaccharide-protein glycosyltransferase subunit STT3, an enzyme that mediates the N-glycosylation of PD-L1, stabilizing its expression on the cell membrane of TNBC cells [11]. The Wnt/β-catenin pathway is also implicated in the regulation of BC metabolism. Specifically, Wnt signalling activation was reported to regulate the expression of pyruvate dehydrogenase kinase, an enzyme that impairs the conversion of pyruvate in acetyl-coA and, thus, is able to block oxidative phosphorylation affecting aerobic glycolysis [30]. In this context, Yang L and colleagues reported the capability of the Wnt/β-catenin responsive gene *c-Myc* to inhibit mitochondrial function in TNBCs as evidenced by reduced mitochondrial DNA and compromised oxidative phosphorylation [31].

### 2.2. Non-Canonical Wnt Signalling

Two different Wnt pathways, defined as “β-catenin-independent”, coexist with the canonical pathway and are generally associated with cell differentiation, polarity, and migration. In the noncanonical pathway, the interaction between Wnt ligands/FZD receptors triggers a series of downstream effectors that mediate the activation of different signalling cascades. In particular, during Wnt/PCP signalling, Wnt ligands bind FZD receptors, which can also cooperate with ROR1/ROR2/RYC proteins, (and activate the small GTPases Ras-related C3 botulinum toxin substrate (RAC) and Ras homologue gene family member A (RHOA) via recruitment and activation of DVL protein [32]. This protein complex stimulates the activity of Rho Associated Coiled-Coil Containing Protein Kinase 1 (ROCK1) and c-Jun N-terminal Kinase (JNK), which, in turn, phosphorylate activating transcription factor 2 (ATF2), leading to rearrangements of the cytoskeleton and triggering the transcriptional activation of target genes responsible for cell adhesion and migration [33] (Figure 1C).

In the Wnt/Ca^2+^ cascade, signalling is initiated by G-protein-triggered phospholipase C (PLC) activity [34], which converts phosphatidylinositol 4,5-bisphosphate (PIP_2_) into diacyl glycerol (DAG) and inositol 1,4,5-trisphosphate (IP_3_). This enzymatic step leads to intracellular Ca^2+^ fluxes and activation of the Ca^2+^-dependent proteins calmodulin kinase 2 (CaMK2) and calcineurin (CaN), whose triggering determines downstream Ca^2+^-dependent cytoskeletal and/or transcriptional responses by nuclear factor of activated T-cell (NFAT) activity [35] (Figure 1D).

### 2.3. Imbalance of the Wnt Pathway in BC

Because mutations in the key intracellular components of the Wnt/β-catenin signalling pathway are rare, identifying the molecular mechanisms of aberrant Wnt activation in BC is critical for the development of activated pathway-targeted therapy. Indeed, considering the role of Wnt signalling in the regulation of different molecular mechanisms of tumour initiation and aggressiveness, many studies highlighted the genetic/epigenetic alterations occurring in Wnt-related gene expression.

#### 2.3.1. Altered Expression of Destruction Complex Components

In this context, genes encoding the proteins constituting the destruction complex were reported to be significantly downregulated in BC. In particular, it has been suggested that disruption of the APC/β-catenin pathway may be involved in breast carcinogenesis. Indeed, Jin Z and colleagues investigated the status of APC gene promoter methylation in primary BCs and in their noncancerous breast tissue counterparts and found that APC expression was epigenetically downregulated in 36% of tested primary BCs through hypermethylation of its promoter and in none of the noncancerous breast tissue samples tested, strongly indicating that APC promoter CpG island hypermethylation represents a cancer-specific change [36]. Of note, the association of aberrant methylation of the APC gene promoter was peculiar to inflammatory BC, a specific histotype characterized by a particularly aggressive outcome disease [37]. Further, in the TNBC subtype, APC expression was also reported to be regulated by the activity of the upregulated miRNA142. Specifically, it was shown that miRNA142 targets the APC transcript, downregulating its transduction [38]. Other pieces of evidence also indicated that lower levels of Axin expression in BC were correlated with higher levels of nuclear β-catenin and may be key in the carcinogenesis and progression of human BC by upregulating the expression of cyclin D1 protein [39].

#### 2.3.2. Altered Expression of FZD/LRP Receptor Complex

In this complex biological scenario, the imbalance of FZD receptor expression is correlated with enhanced tumorigenic activity in BCSCs. Specifically, FZD1 and FZD2 receptors were upregulated in BC cells in comparison with the normal mammary epithelium [40]. Additionally, the abnormal expression of FZD4/5 receptors plays an important role in BC biology since their interaction with WNT10B ligand was found to promote autocrine activation of Wnt signalling in the luminal BC cell line MCF7 [41]. The FZD6 receptor was found to be frequently amplified in BC, with an increased incidence in the TNBC subtype [42]. In particular, deletion of FZD6 expression in BC cell lines inhibited motility/invasion and bone and liver metastasis in xenotransplanted animals. Moreover, multivariate analysis indicated an independent prognostic significance of FZD6 expression in TNBC patients predicting distant, but not local, relapse [42]. Notably, FZD7 expression has also been observed to be altered in the basal-like molecular BC subtype, and its in vitro silencing can significantly impair proliferation and invasiveness coupled with an enhancement of apoptosis in TNBC cells [43]. Another important player in the triggering of the Wnt pathway is the aberrant expression of the LRP6 coreceptor. In particular, it was found to be upregulated in TNBC cell models and in TNBC patients compared with the normal epithelium of the mammary gland, and its silencing was found to reduce Wnt signalling, cell proliferation, and in vivo tumour growth [44]. In this context, BC gene expression profiling of 114 basal-like BC samples significantly associated SOX9, a member of the SOX family of transcription factors and an important mediator of the (epithelial–mesenchymaltransition (EMT) cell program, with the expression of LRP6 and transcription factor 4 (TCF4), two major components of the canonical Wnt/β-catenin pathway. Consistently, SRY-Box Transcription Factor 9 (SOX9) overexpression in BC cell lines and transgenic SOX9 expression in mammary epithelium caused increased LRP6 and TCF4 expression and Wnt/β-catenin activation [45]. Additionally, the expression of Dickkopf-related protein (DKK) family members, which are secreted Wnt signalling antagonists, was found to be altered in BC. Specifically, epigenetic inactivation via promoter methylation of DKK3 was an independent prognostic factor predicting poor overall survival (OS) and short disease-free survival (DFS) in BC [46]. In this context, downregulation of DKK3 was found to promote tumorigenesis of the aggressive basal-like molecular BC subtype [47].

#### 2.3.3. Altered Expression of β-Catenin

β-catenin represents the central node of the canonical Wnt signalling pathway, and its nuclear translocation following the dissociation of the destruction complex is the crucial molecular event for the transcription of Wnt-targeted genes. Specifically, β-catenin was shown to be strictly associated with tumorigenic cell behaviors such as migration, stemness, anchorage-independent growth and chemosensitivity in TNBC models [48]. Furthermore, the increased expression of β-catenin was found to be significantly correlated with the tumour histological grade and Ki-67 labelling, and its expression negatively correlated with BC survival independent of molecular subtype [49]. Moreover, the expression and nuclear location of β-catenin were associated with the initiation and metastasis of spontaneous BC in Tientsin albino 2 mice [50]. Additionally, the coactivators promoting the transcriptional activity mediated by β-catenin were found to be abnormally expressed in BC cells. Specifically, high expression of BRG1, a core component of the SWItch/Sucrose Non-Fermentable (SWI/SNF) chromatin-remodelling complex, was peculiar in TNBC patients with poor prognosis, and its depletion significantly impaired the migration and invasion capability of TNBC cells [51]. Analysis of patient ductal in situ carcinoma (DCIS) revealed a significant correlation between high nuclear BCL9, another coactivator implicated in β-catenin triggering, and pathologic characteristics associated with DCIS recurrence as ER and PgR negative, high nuclear grade, and HER2 amplification/overexpression. Furthermore, in vivo silencing of BCL9 resulted in the inhibition of DCIS invasion and reversal of EMT [52]. Additionally, the aberrant expression of human Pypopus family PHD finger2 (Pygo2), a further mediator of β-catenin activity in the nucleus, was reported to be associated with BC aggressive features, and its altered expression was limited only to BC cells compared with nonmalignant mammary gland compartments [53]. Moreover, Pygo-2 was required for growth in tissue culture and anchorage-independent assays of luminal BC and TNBC cell lines and for the expression of the Wnt target gene Cyclin D1, and its specific silencing was able to significantly impair BC cell proliferation [53].

#### 2.3.4. Altered Expression of Wnt Ligands

Consistent with the role of the main activators of Wnt signalling, different members of Wnt family ligands are directly implicated in BC tumorigenicity and correlated with aggressive disease features. Specifically, the WNT1 ligand is implicated in lobulo-alveolar hyperplasia and ductal branching of the mammary gland [54], and, of note, altered WNT1 expression can promote the insurgence of mammary tumours in transgenic mice engineered to ectopically overexpress its transcript [55]. Moreover, Ayyanan A et al. reported that increased Wnt signalling, as achieved by ectopic expression of WNT-1 in primary human mammary epithelial cells, triggers the DNA damage response and, in turn, a cascade of biological events resulting in their tumorigenic conversion. In addition, WNT-1-transformed cells have high telomerase activity and compromised p53 and Rb function, grow as mammospheres, and in vivo form tumours that closely resemble medullary carcinomas of the breast [56]. Similarly, upregulated WNT3 expression was found to play a key role in the development of resistance to the humanized anti-HER2 monoclonal antibody trastuzumab. Indeed, WNT3 overexpression in trastuzumab-resistant cells increased the nuclear expression of β-catenin, transactivated epidermal growth factor receptor (EGFR), and promoted a partial EMT-like transition, increasing *N*-cadherin, Twist, and Slug and decreasing E-cadherin expression. On the other hand, the knockdown of WNT3 by siRNA restored the cytoplasmic expression of β-catenin and decreased activated EGFR expression in trastuzumab-resistant cells [57]. It was also reported that the elevated expression of WNT5a is associated with malignancy, cell invasion, and metastasis and strongly correlated with the expression of ER. Additionally, high expression levels of WNT5a WNT5a significantly correlated with lymph node metastasis, nuclear grade, and lymphatic invasion, and recurrence-free survival (RFS) was shorter in BC patients with WNT5a expression than in those without [58]. On the other hand, in patients with Estrogen-receptor (ER)-negative disease, lower levels of WNT5a were significantly associated with a worse clinical outcome, consistent with the possibility that the observed function of WNT5a is entirely dependent upon its biological context [59]. Of note, WNT5b was revealed to be a key regulatory factor that governs the phenotype of basal-like BC by activating canonical and non-canonical Wnt signalling, and in turn, its overexpression represents a specific marker of basal-like BC with the worst outcome [60]. In keeping with this finding, another group demonstrated that the silencing of WNT5b expression in TNBC cell lines can significantly impair cell proliferation and invasion [31]. Recently reported results demonstrated that the WNT7b expression level in BC was significantly higher than that in the benign breast, and Kaplan–Meier survival curves demonstrated that patients with high WNT7b expression had shorter overall survival (OS) and RFS than those with low WNT7b expression. In addition, multivariate analysis revealed that WNT7b expression was an independent prognostic factor for both OS and RFS in BC patients [61]. On the other hand, the role of aberrant expression of WNT7a in BC remains unclear. In this context, it was reported that loss of WNT7a expression in BC is associated with poor disease free survival (DFS) but not with poor OS. However, in the ER-positive (ER+) subset, loss of WNT7a expression was an independent prognostic factor for shorter DFS and OS [62]. In keeping with this finding, Avgustinova and colleagues demonstrated that tumour cell-derived Wnt7a recruits and activates fibroblasts to promote tumour aggressiveness both in vitro and in animals [63]. Conversely, in the study published by Chen and colleagues, an abnormal level of WNT7a expression was not associated with aggressive clinicopathologic features and poor clinical outcome of BC patients [61]. To evaluate the extent of WNT ligand modulation in BC cell models, Benhaj et al. reported that most BC cell lines overexpressed members of the WNT ligand family, such as WNT3A, WNT4, WNT6, WNT8B, WNT9A and WNT10B, whereas the expression of WNT5A, WNT5B and WNT16 was usually downregulated. It is important to note that all six WNT ligands that are overexpressed in malignant cell lines are known to signal through the canonical Wnt/β-catenin signalling pathway, whereas downregulated WNT5A and WNT5B ligands signal via the non-canonical pathway [64]. In particular, considering the oncogenic impact of the WNT10B ligand in BC, very recently, the WNT10B network was found to be associated with poor survival and metastases in chemoresistant TNBC [65].

## 3. Wnt Signalling and Resistance to Anti-BC Therapy

As stated above, the altered expression of the different genes involved in the Wnt signalling pathway was largely reported to be implicated in the resistance to chemo/targeted therapies among the different molecular BC subgroups. Specifically, based on data from in vitro cell culture models, the crucial role of EMT in BC resistance to chemotherapy and/or target therapies has been highlighted [66].

### 3.1. Altered Wnt Signaling and Response to Therapy in the HER2+ BC Subtype

In this context, it was reported that upregulation of the WNT3 ligand in HER2-positive (HER2+) BC cells resistant to trastuzumab, the gold standard regimen for HER2+ BC patients, activates the Wnt/β-catenin pathway and promotes the EMT-like transition. Indeed, knockdown of WNT3 by siRNA restored cytoplasmic expression of β-catenin and decreased the expression of EMT markers, suggesting that EMT-like transition may be a unique feature of cells acquiring trastuzumab resistance [57].

Likewise, cyclin-dependent kinase 12 (CDK12), a cyclin-dependent kinase involved in DNA damage repair and coamplified with HER2 at chromosome 17, was reported to play a key role in driving tumorigenesis and inducing anti-HER2 therapy resistance in human BC [67]. Indeed, it was shown that high CDK12 expression is associated with disease recurrence and poor survival and activates WNT1- and WNT2-mediated Wnt/β-catenin signalling cascades to enhance stemness of HER2+ BC and, in turn, to induce trastuzumab resistance [67]. In addition, the Wnt/β-catenin signalling pathway is implicated in the resistance to lapatinib, a tyrosine–kinase inhibitor that potently but reversibly binds to the intracellular tyrosine kinase domain of EGFR and HER2, leading to inhibition of downstream substrate phosphorylation. Specifically, β-catenin knockdown by siRNA significantly increased lapatinib-induced apoptosis in lapatinib-resistant HER2+ BC cells [68].

### 3.2. Altered Wnt Signaling and Response to Therapy in the Luminal BC Subtype

As is well known, the ER+ BC subgroup, characterized by the expression and activity of ER and PgR, represents an optimal target for its antagonist tamoxifen, the gold standard endocrine therapy for these patients. Additionally, in ER+ BC patients, the activation of Wnt/β-catenin signalling was reported to play an important role in tamoxifen resistance [69], and in keeping Loh and colleagues observed that the canonical ER+ BC cell model MCF7, induced in vitro to be resistant to tamoxifen, is characterized by an enhanced EMT phenotype and activation of Wnt signalling via aberrant expression of the WNT3a ligand [70]. Moreover, another group showed that the development of tamoxifen resistance in BC was also driven by sex-determining region y-box2 (SOX2)-dependent activation of Wnt signalling in the BCSC compartment [71]. In addition, the same authors provided evidence that combining hormone therapy and WNT secretion inhibitors might provide a novel strategy to treat BC [71]. In support of this hypothesis, Won et al. suggested that β-catenin plays a key role in tamoxifen-resistant BC and proposed the inhibition of β-catenin as a potential strategy to overcome tamoxifen resistance in ER+ BC [69]. According to the crosstalk between dysregulated Wnt pathway and tamoxifen resistance in ER+ BC, the overexpression of Let-7c miRNA, an oncosupressive miRNA able to inhibit CSC-associated traits, was found to increase tamoxifen sensitivity, impairing the ER-dependent activation of the Wnt signalling pathway [72].

### 3.3. Altered Wnt Signaling and Response to Therapy in the TNBC BC Subtype

Many studies have highlighted the potential implication of Wnt signalling in the onset of chemotherapy resistance in TNBC patients. Accordingly, the TNBC cell line MDAMB231, induced in vitro to be resistant to doxorubicin treatment, relevantly increased the activation of Wnt signalling via enhanced expression of Pygo 2 compared with MDAMB231 wild-type cells [7]. Additionally, the inhibition of Pygo 2 expression was able to revert doxorubicin resistance [7]. Consistent with the implication of Wnt signalling in chemotherapy resistance, El Ayachi and colleagues identified the WNT10B ligand as a key molecule associated with survival and metastases in chemoresistant TNBC [65]. Specifically, WNT10B was unveiled as a biomarker for β-catenin/high-mobility group AT-hook2 (HMGA2)/enhancer of zeste homologue 2 (EZH2) signalling predictive of reduced RFS. Moreover, the silencing of HMGA2 or EZH2 expression or pharmacological inhibition of Wnt signalling in a chemoresistant PDX model of TNBC abolished visceral metastasis, repressing Axin2, Myc, EZH2, and HMGA2 expression in vivo [65]. Additionally, combinatorial therapy of a WNT inhibitor with doxorubicin synergistically activated apoptosis in vitro and resensitized patient-derived xenograft (PDX)-derived cells to doxorubicin, thus repressing lung metastasis in vivo [65]. Moreover, follistatin-like 1 (FSTL1), a secreted follistatin-module-containing glycoprotein that was first identified as a member of the follistatin-SPARC family, was upregulated in TNBC compared with non-TNBC specimens and BC cell lines. In particular, FSTL1 increases oncogenesis in BC by enhancing stemness and chemoresistance to doxorubicin and cisplatin through its ability to activate the integrin β3/Wnt/β-catenin signalling axis in TNBC cells [73].

Furthermore, the Wnt pathway was also linked with the ability of the aryl hydrocarbon receptor (AhR)/cytochrome P450A 1A1 (CYP1A1) pathway to achieve CSC expansion and to mediate chemoresistance in different BC cell models independent of their molecular subtype [74]. Specifically, the AhR/CYP1A1 signalling pathway was found to control BCSC proliferation, development, self-renewal and chemoresistance through inhibition of Phosphatase and tensin homolog (PTEN) and activation of the β-catenin and Akt pathways [74].

## 4. Anti-WNT Compounds in Clinical Trial

In contrast to previous antitumour therapeutic approaches, new treatments need to consider strategic combinations able to kill both CSCs and tumour bulk cells as well as the need to prevent the transition from highly proliferating bulk cells into quiescent CSCs. Considering the crucial role played by the WNT pathway in cancer, the inhibition of WNT signalling represents an interesting and promising therapeutic approach to improve the prognosis of BC patients since its dysregulation is mainly observed in CSCs [75]. Currently, although different types of Wnt/β-catenin pathway inhibitors, such as monoclonal antibodies, recombinant proteins and inhibitors/agonists of Wnt ligands, are under development and in preclinical evaluation in many solid and haematologic malignant models, only a few of them are under investigation in distinct phase I/Ib clinical trials of BC.

### 4.1. Porcupine Inhibitors

LGK974 (ClinicalTrials.gov Identifier: NCT01351103) is a potent and specific small-molecule inhibitor of a porcupine, a membrane-associated acyl-transferase required for the palmitoylation of WNT family ligands, a key step implicated in WNT ligand release [76]. In this context, LGK974 has been reported to halt the secretion of WNT family members outside tumour cells, potently inhibiting Wnt signalling and reducing LRP5/6 phosphorylation and the expression of target genes such as Axin [76]. The antitumour efficacy of LGK974 was primarily tested in transgenic MMTV-WNT1 mice, where it was demonstrated to significantly inhibit tumour growth development [76] and is now under evaluation in a Phase I study of selected patients with different malignancies dependent on Wnt ligands, including TNBC, for whom no effective standard treatment is available.

### 4.2. WNT5a Mimetics

To specifically target the dissemination of BC cells from primary tumours, according to clinical findings showing that loss of WNT5a predicts faster BC spread, a decade ago, Safholm A and colleagues tested their novel WNT5a mimicking hexapeptide, Foxy-5, (ClinicalTrials.gov Identifier: NCT02655952), in preclinical and clinical models as a novel therapeutic strategy to inhibit BC metastasis [77]. In vitro, Foxy-5 treatment demonstrated a significant efficacy to impair the migration and invasion of 4T1 murine mammary tumour cells, and in vivo, following the intraperitoneal injection of 4T1 cells, Foxy-5 administration led to a significant decrease in lung and liver metastasis formation [77]. Their data provided proof of principle that reconstitution of WNT5a signalling represents a valid approach to impair BC metastasis, and the compound is now under evaluation in a clinical Phase I study to establish the recommended dose for a clinical phase II study and enable further development of Foxy-5 as a first-in-class anti-metastatic cancer drug.

### 4.3. FZD Receptors Inhibitors

Wnt pathway inhibition via the targeting of FZD receptors results in decreased growth and tumorigenicity of distinct human oncotypes. In particular, vantictumab (OMP-18R5) (ClinicalTrials.gov Identifier: NCT01973309) is a humanized monoclonal antibody able to bind Frizzled receptors 1, 2, 5, 7 and 8. Vantictumab in combination with paclitaxel resulted in significantly decreased growth and tumorigenicity in BC models [78]. In particular, in xenograft studies, Gurney A. et al. demonstrated that the combination of OMP-18R5 and chemotherapeutic agents reduced tumorigenicity by 10-fold coupled with a parallel decrease in CD44, ALDH1A1, SOX1, and SOX2 genes known to be associated with BCSC frequency and tumorigenicity. In contrast, Taxol treatment in monotherapy significantly increased tumorigenicity [78]. The NCT01973309 trial is a phase Ib clinical trial testing the maximum tolerable dose of vantictumab in combination with paclitaxel in locally recurrent or metastatic BC. The combination of the two compounds demonstrated good tolerability, and of 21 evaluable pts, seven (33%) had a partial response and six (29%) stable disease in the BC metastatic setting [79,80].

### 4.4. Modulating Wnt Pathway in the Clinical Setting

Other molecules that exert indirect activity on the Wnt pathway are currently under investigation for the treatment of BC patients. In this context, the nonsteroidal anti-inflammatory drug celecoxib demonstrated secondary effects on the Wnt pathway. In particular, celecoxib increased the activity of the GSK3β protein, thus enhancing β-catenin segregation in the cytoplasmic compartment in different oncotypes [81,82]. For this reason, the phase III clinical trial REACT (NCT02429427) tested the anticancer effects mediated by celecoxib treatment in comparison with placebo in BC patients. However, REACT failed in its primary and secondary outcomes, and no overall benefit was observed in DFS or OS for celecoxib versus placebo [83].

## 5. Conclusions

The Wnt pathway is complex, with a large number of known ligands, receptors, coreceptors, and regulatory components. Members of the Wnt family have been shown to induce several distinct signalling events involved both in embryo development and homeostasis of tissues, and at the same time, dysregulation of their activity is heavily implicated in tumour initiation and progression, drug resistance and immune escape, crucial features of CSCs. Because Wnt signalling is dysfunctional in various diseases—most importantly, cancer—many efforts are in progress to identify components of the pathway as promising therapeutic targets to eradicate tumour cells, including the minor and critical subset of CSCs, and to select small molecules that interfere with the pathway, ideally in a cell type-specific context. Nevertheless, although the combination of some therapeutic approaches appears promising, many challenges remain to be solved, including potential side effects in NMSCs. The design of effective antitumour strategies able to efficiently target the Wnt pathway could pave the way for dramatic changes in the clinical management of BC patients.
